# Information–Seeking Among Chronic Disease Prevention Staff in State Health Departments: Use of Academic Journals

**DOI:** 10.5888/pcd11.140201

**Published:** 2014-08-14

**Authors:** Jenine K. Harris, Peg Allen, Rebekah R. Jacob, Lindsay Elliott, Ross C. Brownson

**Affiliations:** Author Affiliations: Peg Allen, Rebekah R. Jacob, Lindsay Elliott, Ross C. Brownson, Prevention Research Center in St Louis. Dr Brownson is also affiliated with the Division of Public Health Sciences and Alvin J. Siteman Cancer Center, Washington University School of Medicine, Washington University in St Louis, St Louis, Missouri.

## Abstract

Use of scientific evidence aids in ensuring that public health interventions have the best possible health and economic return on investment. We describe use of academic journals by state health department chronic disease prevention staff to find public health evidence. We surveyed more than 900 state health department staff from all states and the District of Columbia. Participants identified top journals or barriers to journal use. We used descriptive statistics to examine individual and aggregate state health department responses. On average, 45.7% of staff per state health department use journals. Common barriers to use included lack of time, lack of access, and expense. Strategies for increasing journal use are provided.

## Objective

State health departments are key to adoption of evidence-based public health strategies across the public health system (1). Identifying and implementing evidence-based approaches requires access to scientific evidence (2) available through academic journals, conferences, policy briefs, seminars, and elsewhere. The quality of scientific evidence is important; systematic reviews and articles in scientific journals are considered the most objective evidence available (3). We explore state health department staff use of academic journals and barriers to use.

## Methods

All identified state health department professional staff working in chronic disease prevention (ie, tobacco, physical activity, nutrition, obesity prevention, comprehensive cancer, cancer screening, diabetes, heart health) from all 50 states and the District of Columbia (DC) were invited by e-mail to complete an online survey in spring 2013. We identified participants through state health department websites and lists from the Centers for Disease Control and Prevention and National Association of Chronic Disease Directors. After follow-up, 77.3% (904 of 1169 current eligible employees) completed the survey. Test–retest reliability with 75 participants was adequate (Cronbach’s α ≥ .70). Additional survey details are published elsewhere (4); the Washington University in St Louis Institutional Review Board approved this study.

Participants were asked “what methods allow you to learn about the current findings in public health research? (Using the list below please rank the top 3, where 1 is the most important.)” If journals were selected, participants were asked “which journals do you most often read to stay up-to-date on current findings in public health? (Please rank the top 3, with 1 being the journal you most often read.)” If journals were not selected, participants were asked to check all barriers to journal use that applied. Test–retest results showed 62.5% and 91.7% selected 2 or 3 of the same methods and journals, respectively. The barriers question was used in previous research (4).

State health department characteristics were obtained from the Association of State and Territorial Health Officers 2010 survey. We examined the percentage of state health department staff reporting journal use and state health department participation in research activities, staffing levels, mean employee age, mean years at state health department, number of people served, and revenue.

## Results

The 904 respondents were from each of the 50 state health departments and DC. There were 6 to 45 participants per state health department (mean = 31; median = 30). Response rates from state health departments varied from 58.6% to 96.0%. Participants self-identified as program managers or coordinators (57.3%), health educators (12.1%), epidemiologists (8.6%), bureau or division chiefs or directors of chronic disease units (4.5%), and 17.5% other (eg, program evaluators). An average of 45.7% of staff per state health department reported using journals as a top method for finding evidence. State health departments where at least 50% of staff identified journals as a top source participated in more research activities compared with state health departments where fewer than 50% of staff used journals (6.5 activities vs 5.0). There were no other notable differences in journal use for staffing levels, mean employee age, mean years of service, number of people served, or revenue.

**Figure 1:**
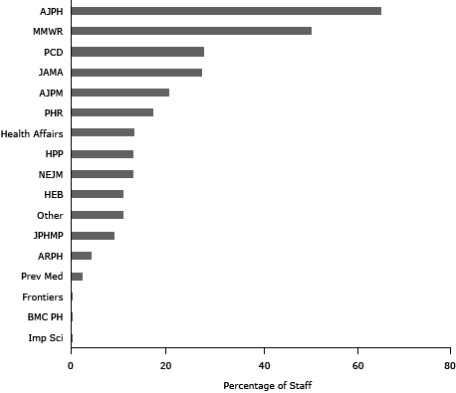
Mean percentage of journal-using staff per health department selecting each journal as 1 of the top 3 they use. **Table:** 

The *American Journal of Public Health* was used by the highest mean percentage of state health department staff (m = 65.4%; Figure 1), followed by *Morbidity and Mortality Weekly Report* (MMWR) (m = 50.8%) and *Preventing Chronic Disease* (PCD) (m = 28.0%). MMWR and PCD are both open-access with a long publication history and affiliation with a national governmental organization.

Journals	Ranking^a^
American Journal of Public Health	.6536
American Journal of Preventive Medicine	.2072
Annual Review of Public Health	.0406
Biomedical Central Public Health (Open Access)	.0022
Frontiers in Public Health Services and Systems Research (Open Access)	.0033
Health Education and Behavior	.1084
Health Promotion Practice	.1295
Implementation Science (Open Access)	.0022
Journal of the American Medical Association	.2746
Journal of Public Health Management and Practice	.0902
Morbidity and Mortality Weekly Report (Open Access)	.5077
New England Journal of Medicine	.1290
Preventing Chronic Disease (Open Access)	.2795
Public Health Reports	.1712
Preventive Medicine	.0219

**Figure 2:**
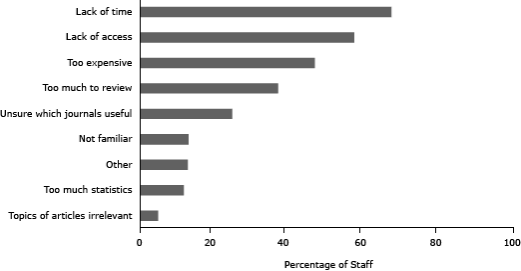
Percentage of non-journal using participants per health department who indicated each barrier to journal use as 1 of their top 3 barriers. **Table:** 

The most common barriers to journal use included lack of time (m = 66.8% per state health department), lack of access (m = 56.9%), and expense (m = 46.4%) (Figure 2).

Barriers to Use of Journals	State Health Department Workers^a ^Who Identified the Barrier, %
Lack of time	.6679
Lack of access	.5691
Too expensive	.4640
Too much to review	.3668
Unsure which journals are useful	.2445
Not familiar	.1289
Other	.1266
Too much statistics	.1165
Article topics are irrelevant	.0479

At the individual level (not aggregated), of those who selected journals as a top source, seminars or workshops were the most highly concurrent method (46.7%) of finding research, followed by policy briefs (32.5%), and e-mail alerts (31.6%). Common methods of finding research for those not selecting journals were seminars or workshops (64.5%), e-mail alerts (57.4%), and newsletters (32.8%).

## Discussion

On average, less than half of staff members per state health department selected journals as a top source of research evidence. Consistent with findings from previous studies (5), common barriers were lack of time, lack of access, expense, and too much to review. Staff members not using journals used seminars or workshops, e-mail, and newsletters. Seminars and workshops address lack of time by providing protected time (6,7), while e-mail and newsletters address too much to review by summarizing information (5). Addressing access is necessary, although likely not sufficient, for increasing journal use. One approach to increasing access, and reducing expense, is developing stronger practice–academia linkages (8,9), which could facilitate formal exchanges of staff (eg, state health department staff as adjunct faculty). The National Network of Libraries of Medicine (NNLM) plans to expand public health digital libraries nationwide (10). Digital libraries can facilitate time savings and promote evidence-based practice for health department staff (10), thus addressing access, expense, and other barriers. Access and expense may also be addressed by increasing availability of open-access journals (11).

Relevance of journal content for state health department practice may also influence use, although relevance was not a top barrier selected. Previous studies of public health agencies found staff wanted access to journals and gray literature for evidence directly related to public health practice (12). Unfortunately, there is limited literature focused on practice; instead, scientific evidence in journals focuses heavily on discovery research (3), which identifies existence of and relationships between health risks and health conditions (eg, smoking and lung cancer) (6,10).

Our main limitation is the use of close-ended measures from previous studies, which may not have captured all existing methods people use to access evidence. Also, “lack of access to journals” was not defined for participants.

State health departments play an important role in protecting and improving public health (1); the use of scientific evidence aids in ensuring public health interventions have the best possible health and economic return on investment (3). Addressing barriers to journal use may increase use of scientific evidence.
